# Untapped ethical resources for neurodegeneration research

**DOI:** 10.1186/1472-6939-12-9

**Published:** 2011-06-02

**Authors:** Julie M Robillard, Carole A Federico, Kate Tairyan, Adrian J Ivinson, Judy Illes

**Affiliations:** 1 National Core for Neuroethics, Division of Neurology, University of British Columbia, Vancouver, BC, Canada; 2 Harvard NeuroDiscovery Center, Harvard Medical School, Boston, MA, USA

## Abstract

**Background:**

The research community has a mandate to discover effective treatments for neurodegenerative disorders. The ethics landscape surrounding this mandate is in a constant state of flux, and ongoing challenges place ever greater demands on investigators to be accountable to the public and to answer questions about the implications of their work for health care, society, and policy.

**Methods:**

We surveyed US-based investigators involved in neurodegenerative diseases research about how they value ethics-related issues, what motivates them to give consideration to those issues, and the barriers to doing so. Using the NIH CRISP database we identified 1,034 researchers with relevant, active grants and invited them to complete an online questionnaire. We received 193 responses. We used exploratory factor analysis to transform individual survey questions into a smaller set of factors, and linear regression to understand the effect of key variables of interest on the factor scores.

**Results:**

Ethics-related issues clustered into two groups: research ethics and external influences. Heads of research groups viewed issues of research ethics to be more important than the other respondents. Concern about external influences was related to overall interest in ethics. Motivators clustered into five groups: ensuring public understanding, external forces, requirements, values, and press and public. Heads of research groups were more motivated to ensure public understanding of research than the other respondents. Barriers clustered into four groups: lack of resources, administrative burden, relevance to the research, and lack of interest. Perceived lack of ethics resources was a particular barrier for investigators working in drug discovery.

**Conclusions:**

The data suggest that senior level neuroscientists working in the field of neurodegeneration (ND), and drug discovery specifically, are motivated to consider ethics issues related to their work, but the perceived lack of ethics resources thwarts their efforts. With bioethics centres at more than 50% of the institutions at which these respondents reside, the neuroscience and bioethics communities appear to be disconnected. Dedicated ethical, legal and social implications (ELSI) programs, such as those fully integrated into genetics and regenerative medicine, provide models for achieving meaningful partnerships not yet adequately realized for scholars and trainees interested in drug discovery for ND.

## Background

Neurodegenerative disease is an umbrella term for illnesses caused by the progressive loss of neurons and their associated functions. This category of diseases includes well known disorders such as Alzheimer's disease, Parkinson's disease and multiple sclerosis, as well as less prevalent diseases such as amyotrophic lateral sclerosis (ALS) and Huntington's disease. The dramatic and debilitating nature of these diseases, the rise in prevalence associated with an aging population, and the general absence of effective treatments [1,2] place them increasingly in the public eye [3]. Despite the failure to discover disease-modifying treatments for most neurodegenerative diseases, the neurobiology research community has in recent years made important advances in the understanding of the root causes of these disorders and possible therapeutic directions [4,5]. There are more active clinical trials ongoing than ever before and many investigators are optimistic that new preventive and therapeutic options will emerge over the next decade [6,7]. As research progresses, public and stakeholder expectations about technology and drug discovery will inevitably intermingle with those of the neuroscience community and generate a variety of ethical issues. It is imperative, therefore, that the societal concerns surrounding potential implications of new findings be integrated into the earliest stages of research. The very nature of neurodegenerative diseases, including their slow but unstoppable progression, the vulnerability of the patient populations, concerns over genetic testing and other diagnostic tools, the prospect that drugs may be used to enhance cognitive function, and the myriad of decisions along the path from bench to bedside makes this field of neuroscience research especially vulnerable to a broad spectrum of ethical issues [8].

Unfortunately, research ethics are commonly misunderstood to be synonymous with administrative burdens rather than understood as a commitment to the moral implications of research [9]. Regulatory requirements poorly aligned to the specific needs of researchers have undermined efforts to integrate ethics into the research process itself. As a result, a significant gap has emerged between laboratory neuroscience research and the societal impact of that research [10].

A growing body of literature addresses the need for neuroscientists to identify and examine the societal implications of their research [11-13]. The relatively new field of neuroethics, at the intersection of biomedical ethics and neuroscience, is aimed at assessing the ethical, legal and social policy implications of research, and is becoming increasingly visible on the international neuroscience scene [14]. As neuroscience plays an increasingly significant role in society, prompting new understandings of people as social and moral beings alongside discoveries of the failures and vulnerabilities of the nervous system, neuroscientists must engage in a discussion of their science and the implications of their work. In the present study, we have characterized the motivators, barriers and priorities for integrating ethics in a cross-section of principal investigators (PIs), faculty members, students and professional staff whose work specifically involves research in neurodegeneration.

## Methods

Using the NIH CRISP database, a searchable database of federally funded research grants of the US-based National Institutes of Health (NIH), and the RePORTER query tool, we identified 1,034 researchers who held active grants with project descriptions containing the key word string "neurodegen*". In order to characterize the most current research activity, we restricted the search to grants awarded between 2007 and 2009. We invited researchers based in the United States who met these criteria to participate in an online survey. Researchers received their invitation by email and were asked to forward the invitation to students, postdoctoral fellows and staff on their research teams. Participation was anonymous and voluntary. All required approvals for this study were obtained from the University of British Columbia Behavioural Research Ethics Board.

The online survey contained 25 open and close-ended questions organized into three sections: I. Ethics in neurodegenerative disease research, II. Motivators and barriers, and III. Current research and background (see Supplementary material). The questions were aimed at characterizing the importance of ethics issues researchers encounter in their work, as well as both motivators and barriers to the inclusion of ethics into neurodegenerative disease research. The identification of factors was guided by prior empirical work on ethics in neuroimaging [15] and finalized in consultation with members of our research team. Answers were either multiple choice or based on a five-point Likert scale, and respondents were encouraged to add free-text narrative content to augment their answers.

To better understand the structure of our data set, we applied exploratory factor analysis to the survey data. This strategy pares down the original large set of survey items into a smaller set of factors, enabling a meaningful interpretation of results. We used exploratory factor analysis with a varimax rotation to ensure that each individual factor can be described by a linear combination of a few functions and analyzed the data separately for each of three categories of ethics-related items: priorities (issues), motivators and barriers. For each category, we retained all factors with a corresponding eigenvalue greater than 1. This process ensures adherence to the Kaiser rule and the appropriate selection of a number of factors that is less than the number needed for perfect reconstruction. We determined sampling adequacy using the Kaiser-Meyer-Olkin (KMO) test to assess the magnitude of partial correlations among variables. For each of the three categories, the KMO test led to a value greater than 0.8; 0.3 greater than the 0.5 required for satisfactory testing. We additionally used Bartlett's method to obtain unbiased estimates of the factor scores for each respondent. These scores were computed as a weighted sum of the original variables. In order to understand the effects of the variables of interest on factor scores, we applied a linear regression model with factor scores as independent variables and variables of interest as predictors.

## Results

### Demographics of the study population

One hundred and ninety three (193) neuroscientists responded to the survey (see Table 1); 62% (119) reported as male and 36% (70) female. Ages ranged between <30 (n = 12, 6%), 31-50 (n = 108, 56%) and 51 and over (n = 65, 34%). A majority of respondents (n = 154, 80%) were at the faculty level. Others were graduate or medical students (n = 17, 9%), postdoctoral fellows (n = 10, 5%) and research staff (n = 5, 3%). A majority of respondents had attained a PhD and/or an MD degree (n = 174, 90%), n = 10 (5%) held a BA or BS equivalent, and n = 7 (4%) held with an MA or MS equivalent. 158 (82%) respondents described themselves as the head of their research group.

**Table 1:** Demographics of study population.

Age	N (%)	Research Area	N (%)
<30	12 (6%)	Drug discovery	97 (50%)

31-50	108 (56%)	Other (e.g., basic research)	80 (41%)

51+	65 (34%)	Regenerative medicine	48 (25%)

**Gender**		**Research Focus**	

Male	119 (62%)	Other (e.g., cell culture)	161 (83%)

Female	70 (36%)	Other animals	134 (69%)

**Professional Level**		Non-human primates	74 (38%)

Faculty	154 (80%)	Adults with ND disease	64 (33%)

Graduate or Medical Student	17 (9%)	Healthy adults	37 (19%)

Post-doctoral Fellow	10 (5%)	**Disease Focus**	

Research Staff	5 (3%)	Alzheimer's	98 (51%)

**Highest Degree Attained**		Other	71 (37%)

PhD and/or MD	174 (90%)	Parkinson's	51 (26%)

BA, BS or equivalent	10 (5%)	Huntington's	38 (20%)

MA, MS or equivalent	7 (4%)	ALS	32 (17%)

**Head of Research Group**		MS	13 (7%)

Yes	158 (82%)		

No	33 (17%)		

Half of the respondent pool indicated that they carried out drug discovery research (50%, n = 97), 25% (n = 48) regenerative medicine research, and 41% (n = 80) classified their type of research as "other" (for example, mechanism of disease and biomarker discovery). Respondents had the opportunity to select more than one type of research.

Participants identified their research subjects as healthy adults (19%, n = 37), adults with neurodegenerative diseases (33%, n = 64), non-human primates (38%, n = 74), other animals (69%, n = 134), or other (for example, cell culture; 83%, n = 161). Disease focus was Alzheimer's (51%, n = 98), Parkinson's (26%, n = 51), Huntington's (20%, n = 38), ALS (17%, n = 32), multiple sclerosis (7%, n = 13), and a range of others (37%, n = 71) (e.g., frontotemporal dementia, prion diseases).

### Ethics-related issues

Exploratory factor analysis revealed that ethics-related issues grouped together under two major factors (Table 2): *research ethics *(accounting for 36.5% of the variance) and *external influences *(accounting for 23% of the variance). The research ethics factor included subject confidentiality (loading of 0.98), privacy (loading of 0.96), and obtaining informed consent (loading of 0.90). The second factor, external influences, included government and public research sponsors (loading of 0.69) and the influence of industry sponsorship on the direction and the topic of the research (loading of 0.67). Regression analysis demonstrated that participants in different research roles attributed different levels of importance to ethical issues: heads of research groups (n = 158) viewed issues surrounding research ethics to be significantly more important than the other respondents (p = 0.01) (Table 3). We also found that overall interest in ethics had the highest effect on concerns about external influences (p < 0.01).

**Table 2:** Exploratory factor analysis: Ethics-related issues, motivators and barriers.

Domain	Clusters of Factors	% of Variance	Cumulative % of Variance	Factor Description	Factor Loadings
*Ethics Related Issues*	Traditional Research Ethics	36.5%	36.5%	Recruiting subjects representing vulnerable populations (a)	0.71
				
				Unrealistic expectations about benefits of the research by subjects (b)	0.65
				
				Subject confidentiality (c)	0.98
				
				Privacy of subjects (d)	0.96
				
				Obtaining informed consent (e)	0.90
				
				Equal access to research for all eligible subjects (f)	0.77
				
				Safety of the method in use (h)	0.62
				
				Clinical findings detected unexpectedly (i)	0.69
	
	External Influences	22.7%	59.2%	Commercial conflict of interest (e.g., timing of technology roll out) (k)	0.63
				
				Priorities of government/public research sponsors (l)	0.69
				
				Influence of industry sponsorship on direction and topics (m)	0.67
				
				Opinion of media and stakeholders (n)	0.63
				
				Opinion of colleagues (o)	0.52
				
				Effect of patents on publication and release of data (p)	0.66

*Motivators*	Ensuring Public Understanding	15.9%	15.9%	Mitigating false hopes or expectations by subjects (m)	0.77
				
				Better informed public and policies (n)	0.75
				
				Patients' right to be informed about neuroscience advances (o)	0.92
	
	External Forces	14.6%	30.5%	Professional advancement (d)	0.78
				
				Institutional encouragement (e)	0.50
				
				Chance of publication success (f)	0.78
				
				Positive perception by clinicians (g)	0.59
	
	Requirements	13.0%	43.5%	Institutional encouragement (e)	0.55
				
				Requirement by the institution where you work (h)	0.99
				
				Requirement by research sponsors (i)	0.69
	
	Values	10.5%	54.0%	Personal values/seems like the right thing to do (a)	0.83
				
				Good citizenship (c)	0.59
	
	Press and Public	8.7%	62.7%	Coverage in the press (k)	0.53
				
				Positive public perception (l)	0.77

*Barriers*	Resources	19.3%	19.3%	Lack of relevant ethics resources (f)	0.92
				
				Lack of access to colleagues with ethics expertise (g)	0.64
	
	Burden	17.4%	36.7%	Increased administrative work (a)	0.72
				
				Lack of time (e)	0.72
	
	Concern	15.7%	52.4%	Ethics is not a relevant or effective tool for my field of research (b)	0.56
				
				Not your job (c)	0.85
	
	Interest	14.8%	67.2%	Lack of individual interest in ethics (h)	0.74
				
				Lack of interest in ethics among neuroscience colleagues (i)	0.67

**Table 3:** Linear regression model: Effect of variables of interest on factor scores.

	Covariates									
	***Gender***	***Subjects***		***Research Type***	***Head of*** ***Research Group***	***Position***		***Interest in Ethics***	***Ethics Consultation***	***Research Goal***

	**Female**	**Non-Human Animals**	**Other** **(e.g., cell culture)**	**Basic**	**Yes**	**Associate Prof/Assistant Prof**	**Postdoc/Grad Student**	**Highly Interested**	**Professional**	**Drug Discovery**

**Factors**										

*Issues*										

Traditional research ethics	-0.05 (p = 0.78)	-0.32 (p = 0.09)	-0.14 (p = 0.67)	-0.06 (p = 0.76)	0.04 (p = 0.94)	**0.47** **(p < 0.01)**	-0.22 (p = 0.70)	0.15 (p = 0.36)	**0.39** **(p < 0.05)**	-0.00 (p = 0.99)

External forces	-0.30 (p = 0.13)	0.17 (p = 0.41)	-0.02 (p = 0.96)	-0.23 (p = 0.32)	0.72 (p = 0.19)	-0.25 (p = 0.22)	0.44 (p = 0.49)	**0.64** **(p < 0.01)**	-0.27 (p = 0.17)	0.15 (p = 0.43)

*Motivators*										

Ensuring public understanding	**0.55** **(p < 0.01)**	-0.36 (p = 0.06)	**-0.67** **(p < 0.05)**	-0.03 (p = 0.87)	0.84 (p = 0.07)	0.14 (p = 0.44)	0.57 (p = 0.30)	0.20 (p = 0.24)	0.24 (p = 0.18)	0.26 (p = 0.13)

External forces	-0.11 (p = 0.58)	-0.01 (p = 0.97)	-0.08 (p = 0.83)	0.31 (p = 0.22)	-0.05 (p = 0.93)	0.36 (p = 0.10)	0.56 (p = 0.36)	0.05 (p = 0.79)	0.03 (p = 0.89)	0.03 (p = 0.88)

Requirements	-0.07 (p = 0.70)	-0.15 (p = 0.41)	0.01 (p = 0.97)	0.19 (p = 0.37)	-0.87 (p = 0.06)	0.29 (p = 0.12)	-0.36 (p = 0.51)	-0.06 (p = 0.72)	0.24 (p = 0.19)	-0.00 (p = 0.99)

Values	0.15 (p = 0.44)	0.14 (p = 0.53)	0.17 (p = 0.66)	-0.13 (p = 0.59)	-0.54 (p = 0.31)	0.01 (p = 0.96)	-0.54 (p = 0.39)	**0.41** **(p < 0.05)**	0.06 (p = 0.78)	-0.15 (p = 0.45)

Press and public	-0.15 (p = 0.46)	0.10 (p = 0.66)	**-1.02** **(p < 0.01)**	-0.21 (p = 0.39)	0.17 (p = 0.75)	-0.18 (p = 0.40)	0.18 (p = 0.78)	-0.00 (p = 0.99)	0.14 (p = 0.51)	-0.02 (p = 0.91)

*Barriers*										

Resources	0.07 (p = 0.72)	-0.18 (p = 0.35)	-0.19 (p = 0.60)	0.11 (p = 0.59)	-0.01 (p = 0.99)	-0.22 (p = 0.25)	0.20 (p = 0.72)	0.24 (p = 0.15)	0.20 (p = 0.27)	0.34 (p = 0.06)

Burden	-0.12 (p = 0.57)	**-0.52** **(p < 0.05)**	-0.48 (p = 0.27)	0.11 (p = 0.65)	-0.10 (p = 0.85)	-0.05 (p = 0.83)	-0.54 (p = 0.41)	-0.12 (p = 0.55)	0.22 (p = 0.31)	0.02 (p = 0.92)

Concern	**-0.57** **(p < 0.01)**	**0.48** **(p < 0.05)**	0.21 (p = 0.59)	0.09 (p = 0.69)	-0.39 (p = 0.43)	0.09 (p = 0.65)	-0.80 (p = 0.18)	-0.35 (p = 0.06)	**-0.54** **(p < 0.01)**	0.16 (p = 0.41)

Interest	-0.13 (p = 0.56)	0.05 (p = 0.84)	0.05 (p = 0.90)	0.07 (p = 0.80)	0.72 (p = 0.21)	0.31 (p = 0.17)	0.78 (p = 0.25)	-0.33 (p = 0.11)	0.13 (p = 0.55)	0.16 (p = 0.46)

Open-ended comments suggested that communication of results represents an additional ethical concern in neurodegenerative disease research. One investigator commented on science communication in the media: "Media usually provide uneducated information to (the) public" [Respondent #32]. In terms of disseminating results specifically to stakeholders, another researcher commented on the difficulty of assessing what, when and how to disclose research results to subjects: "Paternalism in withholding versus difficulty (in) accurately presenting scientific uncertainty" [Respondent #45]. Several researchers (n = 21) commented on issues of ethics in animal care: "(...) the ethical care and use of animals is important" [Respondent #21]. Open-ended narrative responses also indicated that conflict of interest represents an ethical issue in the field of neurodegenerative research, but opinions about this varied. For example, one respondent indicated that the "...pendulum has now swung too far at some academic institutions on conflict of interest rules. We must now anticipate a year in advance what might possibly maybe lead to a financial interest." [Respondent #11]. Another wrote an opinion to the contrary, on the issue of "authors not adequately disclosing potential conflict of interests" [Respondent #150].

### Ethics-related motivators

Ethics-related motivators grouped under five factors (Table 2). The first, *ensuring public understanding *(accounting for 15.9% of the variance), included a person's right to be informed about neuroscience advances (loading of 0.94) and mitigating false hopes or expectations by research subjects (loading of 0.77). The second factor, *external forces *(14.6% of the variance), included the motivators of professional advancement (loading of 0.78), chance of publication success (loading of 0.78), and institutional encouragement (loading of 0.5). The third factor, *requirements *(13% of the variance), included obligations to the research institution (loading of 0.99) and to research sponsors (loading of 0.69). The fourth factor, *values *(10.5% of the variance), included items such as personal values, and the feeling that being concerned with ethics is "the right thing to do" (loading of 0.83). The fifth and final factor was *press and public *(8.7% of the variance), and included motivators related to coverage in the press (loading of 0.53) and positive public perception (loading of 0.77).

Regression analysis highlights an important and significant effect for professional seniority: heads of research groups were more motivated to ensure public understanding of their research than the other respondents (p < 0.01) (Table 3). On this same factor of public understanding of research, we noted that female researchers rated the importance of matters of public understanding of research more highly than male researchers (p = 0.02). Other motivators included both factors that tie in with public opinion, such as "Increase in donations" [Respondent #29] as well as factors that tie in with the implications of the research for society, such as "Influence on legal developments and regulations" [Respondent #140]. Two respondents also highlighted a personal motivation to consider ethics in research: "... own dedication to trying to cure diseases" [Respondent #152].

### Ethics-related barriers

Barriers to the consideration of ethics in neurodegenerative disease research grouped under four factors: *resources *(lack of resources and expertise, accounting for 19.3% of the variance), *burden *(lack of time and increased administrative work; 17.4% of the variance), *concern *(ethics are not relevant to the research; 15.7% of the variance) and *interest *(lack of interest in ethics from researcher and colleagues; 14.5% of the variance). A salient significant finding from the regression analysis with these factors is that a perceived lack of ethics resources is a barrier for investigators working in the area of drug discovery (p < 0.05). The open-ended comments about ethics-related barriers contributed by respondents spoke to both the perceived fundamental nature of bioethics, as one respondent wrote: "Teaching bioethics sometimes seems like teaching right from wrong" [Respondent #122] as well as to the practical implications of incorporating ethics into research: "(There is a) lack of understanding by IRB (...) members regarding practical limitations and time tables that impact research" [Respondent #131].

## Discussion

The data presented here suggest that senior level neuroscientists working in the field of neurodegenerative diseases are motivated to consider ethics issues related to their work. In addition, these researchers are motivated to ensure public understanding of their research. However for researchers specifically in the area of drug discovery, we found that a perceived lack of ethics resources is a significant barrier to pursuing these interests and goals.

These findings closely mirror those of a related study by our group carried out in the field of neuroimaging [15]. Data from that study and others suggest that researchers see ethics as complex, overregulated, and overly time consuming [15,16]. However, considerable differences also exist between our neurodegeneration research study and the neuroimaging study. Neuroimaging trainees rate indifference as a significant barrier, as well as a lack of ethics resources. In contrast, we find that in neurodegeneration research, the lack of ethics resources acts as a barrier for researchers in drug discovery regardless of professional level. Similarly, gender effects were present in both cohorts of researchers but differ in valence. The data from the present study suggest that female researchers rate matters of public understanding of research more highly than male researchers. In the neuroimaging study, female researchers rated issues such as recruitment, confidentiality and privacy of human subjects more highly than male researchers, tended to value trust and reciprocity more than male researchers, and considered indifference to be less of a barrier than the male researchers. There we speculated that the source of the effect may be associated with the leadership and organizational skills of women that underscore the importance of positive group dynamics and mutual respect [17]. This is explanation may apply to the present results as well, although more study of this phenomenon is clearly needed. There is also some evidence for a distinctively feminine moral voice. According to Gilligan, whereas the typical male moral voice speaks the language of justice, rights, and rules, the female moral voice speaks a language of care that emphasizes relationships and responsibilities [18]. These findings are controversial, but could explain the gender effect observed.

Finally, while both cohorts represented North American investigators involved in neuroscience research, our data also suggest that differing ethical issues may exist for different subspecialties and present different barriers to the consideration of ethics in the appropriate research context.

We recognize the limitations of the study. Our response rate was 19%. The sample was not random and was limited to investigators holding government grants in the United States. As well, responses likely reflect the views of researchers with a pre-existing interest in ethics. These characteristics limit the generalizability of our results to the broader community of neuroscience researchers. We also acknowledge that some of our survey questions were tailored to investigators who work with human subjects, and therefore may not have been appropriate to our entire sample. In particular, investigators who work with animals identified animal welfare as an additional ethical consideration, an issue that we plan to address in future work. Finally, our current work does not deliver a quantitative measure of the degree to which the issues and barriers identified impact research. Future studies of larger cohorts and in-depth interviews with researchers will serve to expand our findings and will provide a more detailed ethical landscape for neurodegeneration research.

## Conclusion

While is it worthwhile to identify the barriers and the motivators for the consideration of ethics in neuroscience research, it is equally important to propose solution-oriented strategies to address the issues uncovered. This is especially relevant in the face of the growing prevalence of age-associated neurodegenerative diseases and the growing need for new approaches to diagnose, treat and prevent these conditions. We agree with Samarasekera [19] who, in a recent opinion piece, encouraged the development of meaningful partnerships between neuroscientists and ethicists. As junior scientists today represent the investigators of tomorrow, it is also essential to develop and integrate relevant ethics curricula into graduate training [20]. Finally, the formal ethics review process would greatly benefit from enhanced communication channels between the institutional review boards and ethics committees and the investigators themselves. Improved communication between ethicists and neuroscientists beyond the institutional review board requirements will lead to a better understanding of the ethics needs of the research community and have a positive impact on research conduct, public understanding of science and, ultimately, public policy.

## Competing interests

The authors declare that they have no competing interests. They declare themselves to be independent of funders.

## Authors' contributions

JR participated in the interpretation of the data and authored the first draft of the manuscript. JI and AJI conceived of the study, and participated in its design and coordination and helped to draft the manuscript. KT and CF carried out the survey, participated in the analysis of the data, and helped to draft the manuscript. All authors read and approved the final manuscript.

## Pre-publication history

The pre-publication history for this paper can be accessed here:

http://www.biomedcentral.com/1472-6939/12/9/prepub
